# Using reflectance to measure chlorophyll *a* in corals: calibration and implications of skeletal optical properties

**DOI:** 10.1007/s00338-026-02831-0

**Published:** 2026-02-17

**Authors:** Kay Watty, Verena Schoepf, Kelly W. Johnson, Sophie Littke, Rene M. van der Zande

**Affiliations:** 1 Department of Freshwater and Marine Ecology, Institute for Biodiversity and Ecosystem Dynamics, University of Amsterdam, Amsterdam, The Netherlands; 2 University of Algarve, Faro, Portugal

**Keywords:** Coral bleaching, NDVI, Reflectance, Chlorophyll *a*, Light scattering, Symbiodiniaceae

## Abstract

Climate change-driven coral bleaching and associated chlorophyll (Chl) loss increasingly threaten coral reefs, highlighting the need for non-invasive, cost-effective tools to assess corals’ physiological condition. Researchers have explored the reflectance-based Normalized Difference Vegetation Index (NDVI) as a proxy for Chl *a*. However, almost no calibration curves exist to date; thus it remains unknown if the relationship between Chl *a* and NDVI differs among coral species, and whether it is influenced by skeletal optical properties. Here, we correlated Chl *a* content to NDVI in three coral species with different skeletal morphologies and reflectance modes across a range of health states (i.e., bleaching states). We found a strong positive relationship between Chl *a* and NDVI in all species, validating NDVI as a proxy for Chl *a* in corals. However, this relationship is non-linear, showing deviations at the highest and lowest ends of the pigmentation spectrum. We propose that in healthy states, this non-linearity is due to pigment self-shading, whereas in bleaching states, it is caused by backscattering of light by the increasingly exposed coral skeleton, because pigment loss enhances absorption by the remaining Chl *a* pigments and lowers, rather than increases, reflectance. To avoid Chl *a* under- or overestimation, we recommend to log-transform Chl *a* and create species-specific calibration curves. Overall, the non-invasive, repeatable, and scalable nature of reflectance measurements and NDVI make them valuable tools for assessing coral health both ex- and in situ. They provide a comprehensive view of the holobiont’s physiological condition and hold potential for broader applications in reef-scale monitoring, remote sensing, coral restoration, and conservation.

**Supplementary Information:**

The online version contains supplementary material available at 10.1007/s00338-026-02831-0.

## Introduction

Climate change poses a growing threat to coral reef ecosystems, with marine heatwaves becoming more frequent and intense (Oliver et al. 2018; Lough et al. 2018). Elevated temperatures are the primary driver of coral bleaching, disrupting the symbiosis between algal symbionts (Symbiodiniaceae) and their coral host, often leading to symbiont loss. Since the symbionts provide much of the coral’s daily energetic demand through photosynthesis, their loss leaves corals energetically starved and visibly pale, with the white carbonate skeleton becoming exposed (e.g., Brown 1997; Hoegh-Guldberg 1999). In times of unprecedented coral loss, destructive sampling should be avoided as much as possible. However, non-invasive, rapid, and high-throughput phenotyping tools to assess the physiological condition of corals remain limited (Dellisanti et al. 2021; Davies et al. 2023).

Conventionally, coral fragments are sacrificed, pigments extracted with a solvent, and absorbance measured spectrophotometrically to quantify the chlorophyll (Chl) within the endosymbiotic dinoflagellates as a measure of coral bleaching. Because Chl *a* is the dominant photosynthetic pigment in symbiotic dinoflagellates (Jeffrey and Haxo 1968), responds strongly to stress, and reliably predicts photosynthetic performance (Anthony et al. 2009), studies have primarily focused on quantifying Chl *a* (Hochberg et al. 2006). However, besides being destructive, conventional methods provide data only for limited time points and little information about the progression of bleaching. To overcome these limitations, other standardized methods have been applied, such as RGB or grayscale image analysis (e.g., Chow et al. 2016; Winters et al. 2009; Ferrara et al. 2024) and the Coral Health Chart (Siebeck et al. 2006). While useful, these offer low resolution in detecting optical changes, or rely on subjective assessment. In terrestrial plant research, the Normalized Difference Vegetation Index (NDVI) is a widely used reflectance-based proxy for Chl *a* (Rouse et al. 1973). NDVI has recently been explored as a non-invasive method for corals as well (Rocha et al. 2013; Wijgerde et al. 2014; Leal et al. 2015; Denis et al. 2024; Naugle et al. 2024, 2025; Veeranjaneyulu et al. 2024), though its use in marine ecology remains limited. The index is based on the different light absorption features of Chl *a* in the red and near-infrared spectrum, and it can be calculated rapidly, and at low cost from spectral reflectance measurements. High Chl *a* concentrations cause low reflectance due to high light absorption, leading to high NDVI values, and vice versa (Leal et al. 2015).

To establish NDVI as a reliable proxy for Chl *a*, it must show a strong correlation with measured Chl *a* across species and conditions, using standardized spectral methods. While Leal et al. (2015) demonstrated a clear linear relationship between NDVI and Chl *a* content in the soft coral *Sarcophyton* cf. *glaucum* with a strong regression model fit (R^2^ = 0.9), a somewhat different and weaker relationship was reported for two *Acropora* species (Naugle et al. 2024; Denis et al. 2024). Although they showed significant correlations that support the relationship between NDVI and Chl *a*, their data distributions deviated from linearity, raising crucial questions about whether skeletal scattering alters the reflectance signal and, thus, the validity of using NDVI as a reliable and accurate proxy for Chl *a* in scleractinian corals.

Coral skeletons are known to scatter light diffusely, i.e., reflecting it equally in all directions, and altering the internal light environment for the symbionts (Kühl et al. 1995; Enríquez et al. 2005; Terán et al. 2010; Wangpraseurt et al. 2012). Because coral tissue is largely translucent, light easily reaches the underlying skeleton. The optical microenvironment of corals is thus mainly governed by the density of the endosymbiont pigments, as well as the optical properties of the coral tissue and skeleton (Wangpraseurt et al. 2012; Bollati et al. 2020). As pigment density changes due to bleaching, it alters how much light reaches the skeleton and is subsequently scattered. Bleached corals typically display a higher reflectance as the underlying calcium carbonate skeleton becomes exposed (Yamano et al. 2003; Scheufen et al. 2017), resembling the spectral properties of carbonate sand (Hochberg et al. 2003). The decline in Chl *a* reduces NDVI, as characteristic absorption features diminish (Terán et al. 2010). However, increased exposure of the underlying skeleton can interfere with the reflectance measurements, as the absorption efficiency of the remaining Chl *a* pigments may change when the exposed skeleton scatters more light (Enríquez et al. 2005; Terán et al. 2010). This is an artefact of coral skeletal structure that was not a factor in the original development of NDVI for terrestrial vegetation.

Spectral reflectance has already been a central parameter to coral reef remote sensing, used to characterize the bottom-types of benthic communities and establish spectral reflectance modes of corals (Hochberg et al. 2003, 2004). With the advancement of hyperspectral cameras and the newly introduced MINI-SPEC spectrometer being available as part of the DIVING-PAM-II (Walz GmbH, Germany), NDVI can be expected to become increasingly employed in coral reef research. However, few calibration curves exist as the relationship between NDVI and Chl *a* has only been established in one soft and two hard coral species to date (Leal et al. 2015; Naugle et al. 2024; Denis et al. 2024). It is therefore poorly understood if this relationship is species-specific and how it is influenced by skeletal optical properties.

To address this knowledge gap, we exposed three coral species with different skeletal morphologies and reflectance modes to heat stress to produce a range of health (or bleaching) states. We then measured spectral reflectance on the live corals and later quantified Chl *a* pigment content to investigate the underlying relationship between NDVI and Chl *a* across a range of pigment concentrations and establish calibration curves. We hypothesized that the relationship between NDVI and Chl *a* in scleractinian corals is strong, but non-linear, and that this pattern reflects species-specific optical skeleton traits and pigment dynamics. Understanding this relationship is critical for developing NDVI into a robust, non-invasive, rapid, and high-throughput phenotyping tool for assessing corals’ physiological conditions.

## Methods

### Sample collection

**Fig. 1:**
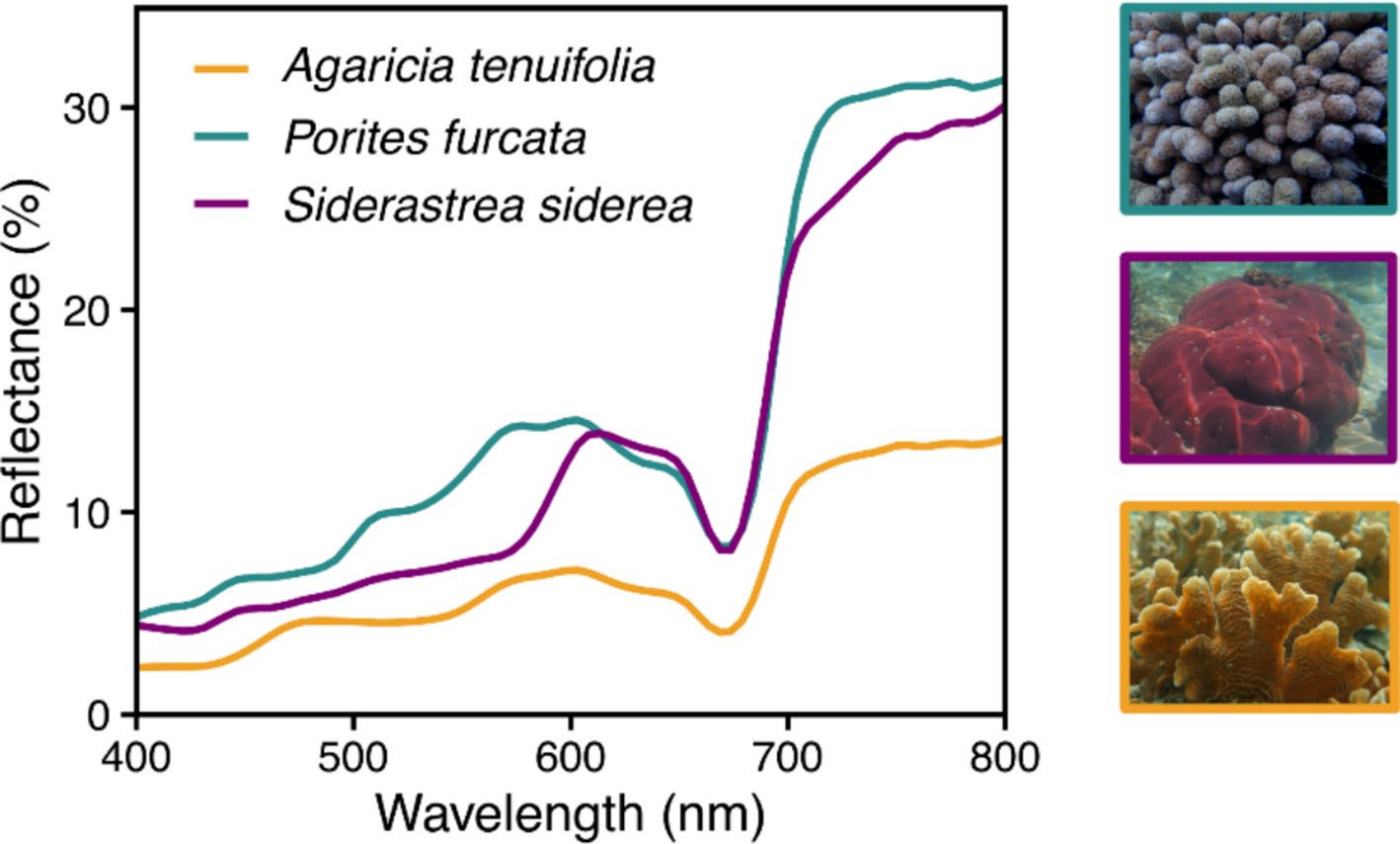
Spectral reflectance of the Caribbean coral species *Porites furcata* (teal, n = 112), *Siderastrea siderea *(purple, n = 56), and* Agaricia tenuifolia* (yellow, n = 51). Reflectance profiles represent mean reflectance of corals per species prior to the heat-stress treatments. Note that *A. tenuifolia* and *P. furcata* have different reflectance peaks around 575 nm compared to *S. siderea*, highlighting the brown and blue coral modes, respectively. The clear minimum in reflectance around 670 nm for all corals reflects the absorption maximum of chlorophyll *a* in the red region of the PAR spectrum. Photo credit: V. Schoepf, K.W. Johnson, and S. Littke

The three Caribbean coral species *Agaricia tenuifolia*, *Porites furcata*, and *Siderastrea siderea* were used to investigate the relationship between Chl *a* content and NDVI. Scleractinian corals can be broadly categorized by their spectral reflectance into brown and blue modes (Hochberg et al. 2004), and the species studied here represent both groups: *A. tenuifolia* and *P. furcata* fall into the brown mode, while *S. siderea* represents the blue mode (Fig. 1). *Porites furcata* (n = 112 fragments from 15 colonies) was collected in January 2024 from Director’s Bay Reef in Curaçao (research permit 23CW021 issued by the Ministry of Health, Environment and Nature, Curaçao), transported to the Netherlands (CITES permit 24NL322285/11), and held in indoor aquaria at Blijdorp Zoo in Rotterdam for 12 weeks prior to heating treatments. *Agaricia tenuifolia* (n = 51 fragments from 6 colonies) and *S. siderea* (n = 56 fragments from 6 colonies) were collected in September 2024 from Bahía Almirante in Bocas del Toro, Panama under research permit ARB-096–2023 issued by Ministerio de Ambiente, Panama. These corals were held in aquaria at the Smithsonian Tropical Research Institute in Bocas del Toro, Panama during heating treatments, and eventually exported under CITES permit PA01ARB190-2024. Species identification was based on colony morphology and skeletal characters. All corals were collected from a 3–6 m depth range.

### Experimental setup

Coral species were separately subjected to heating treatments over several days to weeks to induce bleaching and generate a broad range of Chl *a* concentrations to correlate with spectral reflectance-derived NDVI. *Porites furcata* fragments were subjected to 31.7 ± 0.5 °C and exposed to an average daily light intensity of ∼320 μmol photons m^−2^ s^−1^ (LED lights, Zeus, Ledzealer, China), with a 12 h light:12 h dark photoperiod and a peak of 525 μmol photons m^−2^ s^−1^ at noon, over a 14 day heat period. *Agaricia tenuifolia* and *S. siderea* were exposed to an average temperature of 30.1 °C for 21 days. At the end of these respective periods, reflectance measurements were taken of all fragments for NDVI determination. Fragments were subsequently frozen at −20 °C for Chl *a* extraction and correlation with NDVI calculated for the end-point reflectance measurement.

### Spectral reflectance

To provide a proof-of-principle and show that pigment changes can be tracked over time using NDVI, spectral reflectance (*R*) of independent *P. furcata* fragments (subset of n = 14) was measured every other day in the second week of the heat stress period. To correlate NDVI and Chl *a*, *R* was measured on *A. tenuifolia* (n = 51), *S. siderea* (n = 56), and *P. furcata* (n = 112) fragments representing different bleaching states using the MINI-SPEC spectrometer of the DIVING-PAM-II (Walz GmbH), followed by immediate freezing of the fragments at −20 °C for Chl *a* extraction. Reflectance measurements were taken underwater to avoid artifacts caused by the water surface or by wet coral tissue (see Johnsen 2016) and were standardized using the provided Spectralon white standard. NDVI was calculated following Eq. 1:

1 $$\begin{array}{c}NDVI= \frac{{R}_{750}-{R}_{670}}{{R}_{750}+{R}_{670}} \end{array}$$

Here, *R*_670_ and *R*_750_ represent the mean reflectance in the red and near-infrared region of the electromagnetic spectrum, respectively (adapted from Rocha et al. 2013). Due to potential differences in areal Chl *a* concentrations across a single fragment, measurements were taken on three randomly selected points per coral fragment and *R* averaged per wavelength for the calculation of NDVI to obtain a single average value for NDVI representing an entire fragment (for the detailed protocol, see Watty et al. 2024).

### Chlorophyll *a* quantification

Chl *a* content was measured for each coral fragment to quantify pigment concentrations across the different bleaching states. Upon thawing, the coral tissue was separated from the skeleton using an airbrush and 25 ml Milli-Q water. For *S. siderea*, 50–400 ml of Milli-Q water were used with a waterpik in addition to the airbrush. The slurry mixture was centrifuged (RCF: 3220 $\times$ g) to mass-separate the mixture into coral (supernatant) and symbiont (pellet) fractions (Eppendorf Centrifuge 5810 R). After discarding the supernatant, the pellet was resuspended in 4 ml Milli-Q and centrifuged again in order to remove coral mucus, for a total of three washes. Finally, chlorophylls were extracted from the clean pellet using 1.5 ml 100% acetone. After 24 h of dark-extraction at -20 °C, the absorbance of the solution was measured at 630, 663, and 750 nm using a spectrophotometer (Novaspec Pro, Biochrom, USA), with 100% acetone as the blank. Chl *a* concentrations were calculated based on the equations from Jeffrey and Humphrey (1975) and standardized to extraction volume. Fragment surface area was established using 3D photogrammetry (Ferrari et al. 2016). In summary, 40–50 photos per fragment including size reference were taken from all directions at 0°, 45° and 90° angles. Photos were then processed using Autodesk Recap Photo software (Autodesk, California, USA) into 3D models. Models were then calibrated and trimmed to represent only live coral tissue, after which total surface area was extracted.

### Absorption coefficient of chlorophyll *a*

To investigate changes in light absorption by Chl *a* during bleaching, the absorption coefficient (a*) was calculated following Enríquez et al. (2005). This metric reflects how efficiently pigments absorb light relative to their concentration. First, the absorbance (*D*) specific to Chl *a* was derived from *R* at 670 nm using Eq. 2:

2 $$\begin{array}{c}{D}_{670 }=\mathrm{log}\left(\frac{1}{{R}_{670}}\right)\end{array}$$

Subsequently, the Chl *a*-specific absorption coefficient (a*_Chl* a*_) was computed by normalizing *D*_670_ to the pigment content per surface area (*p*) using Eq. 3 (sensu Enríquez et al. 2005):

3 $$\begin{array}{c}{{a}^{*}}_{\text{Chl }a}=\left(\frac{{D}_{670}}{p}\right)ln10\end{array}$$

### Data analysis

The relationship between Chl *a* content and NDVI was analyzed by fitting three models: a linear regression on the untransformed Chl *a* data, a linear regression on log-transformed Chl *a*, and a non-linear least squares exponential model of the form $y=a {e}^{bx}$. Model performance was compared using R^2^ values and the Akaike Information Criterion (AIC). In addition, Spearman and Pearson correlation coefficients were calculated to assess non-linear (monotonic) and linear relationships between NDVI and Chl *a*, respectively. The linear regression on log-transformed Chl *a* was back-transformed and plotted over the raw data to further investigate non-linearity in the relationship. Diagnostic plots were generated for each model to assess assumptions of normality and homoscedasticity across fitted levels. Cook’s distance was used to assess the influence of individual observations on fitted models (see Supplementary Methods for details). Additionally, Shapiro–Wilk tests were performed to formally test the normality of residuals. The decline of a*_Chl *a*_ with increasing Chl *a* concentration was modeled using a non-linear least squares exponential model. All analyses were conducted in R (version 4.4.1; R Core Team, 2024).

## Results and discussion

**Fig. 2:**
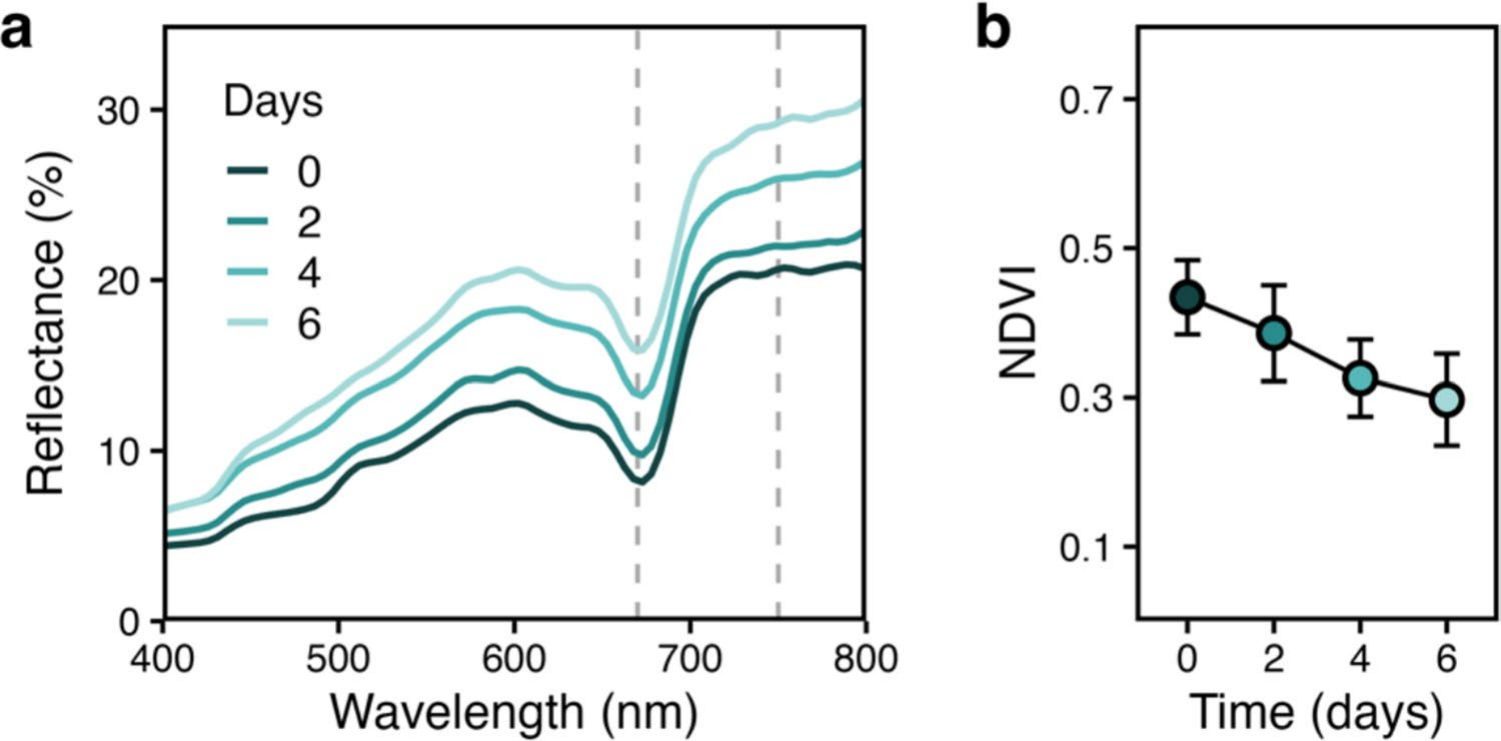
Spectral changes over the progression of coral bleaching in *Porites furcata*, measured on a subset of 14 fragments. **a **Mean relative reflectance of coral surfaces over 400-800 nm standardized to a Spectralon white standard. Days 0–6 represent measurements taken at two-day intervals during the second week of a 14-day thermal stress period, highlighting a gradual increase in reflectance, and concurrent decline in NDVI with bleaching progression. Vertical dashed lines indicate the wavelengths used to calculate NDVI (670 and 750 nm). **b** NDVI calculated as a proxy for Chl *a* over progressing time points (Days 0–6) during the heat-stress treatment (mean ± se).

**Fig. 3:**
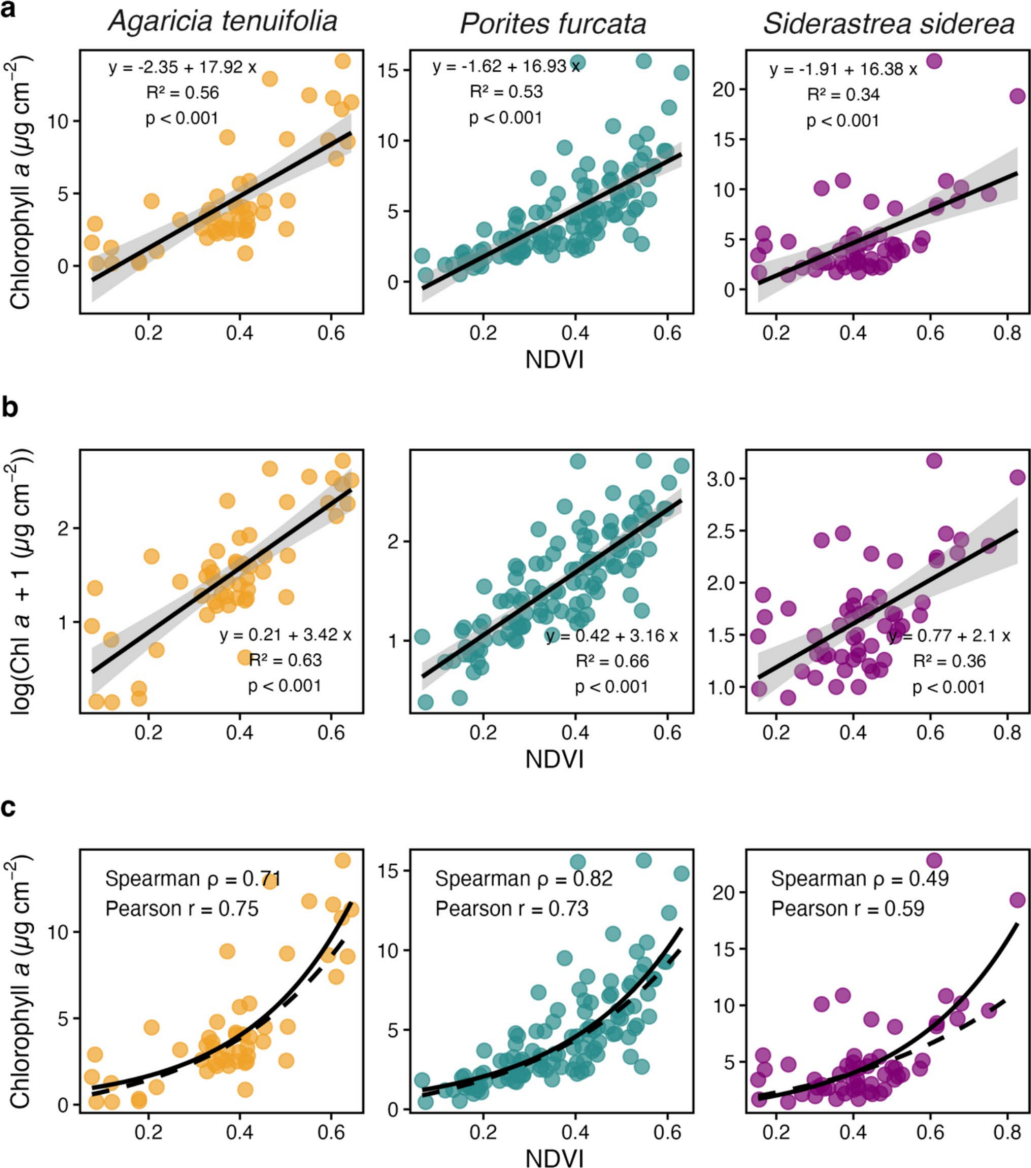
Relationship between chlorophyll *a* (Chl *a*) concentration (µg cm^-2^) and NDVI for *Agaricia tenuifolia* (yellow), *Porites furcata* (teal) and *Siderastrea siderea* (purple). **a** Raw Chl *a* concentrations plotted against NDVI, faceted by species. Points represent individual coral fragments; solid lines show fitted linear regression models with equations, effect sizes, and significance shown in each panel. **b** Log-transformed Chl *a* concentrations against NDVI, with linear regression models per species and corresponding statistical details displayed. **c** Raw Chl *a* concentrations with fitted exponential models (solid lines) and back-transformed log-linear models (dashed lines) per species. Spearman's $\rho$ and Pearson’s *r* are shown to indicate monotonic and linear associations, respectively. Model functions are provided in Table S4.

During bleaching spectral reflectance shifted upwards and NDVI declined, as shown for *P. furcata* under heat stress (Fig. 2). This proof-of-principle shows that NDVI effectively captures pigment loss in corals over time. Analysis of model characteristics for the calibration curves highlights a consistent, but species-specific association between spectral reflectance-derived NDVI and Chl *a* content. Spearman and Pearson correlation analyses revealed a significant positive association between NDVI and Chl *a* content across all species, indicating strong monotonic relationships (Table S1). Regression models were highly significant across species and models (p < 0.001). Of the three models evaluated, the linear regression on log-transformed Chl *a* provided the best fit across species (Fig. 3; Table S2), achieving best scores for both R^2^ and AIC in *P. furcata*. Its back-transformation closely resembled the non-linear exponential fit, indicating that the underlying relationship between NDVI and Chl *a* is likely exponential*.* In *A. tenuifolia* and *S. siderea*, the linear model fit on log-transformed Chl *a* had a much lower AIC compared to the exponential model, indicating a better balance between model complexity and fit, despite a slightly higher R^2^ for the exponential model. Shapiro–Wilk tests of model residuals (Table S3) and diagnostic plots (Supplementary Figs. S1-S3) further supported these trends. Together, these findings corroborate the strong correlation between NDVI and Chl *a* reported for soft and hard corals in the literature (Leal et al. 2015; Denis et al. 2024; Naugle et al. 2024) and show that this relationship holds across species representing different spectral modes (Hochberg et al. 2004). In addition, calibration curves for five scleractinian coral species (this study, Naugle et al. 2024; Denis et al. 2024) show that the relationship between NDVI and Chl *a* in hard corals is best captured when log-transforming Chl *a*, likely due to underlying non-linear patterns (see Supplementary Results for more details).

**Fig. 4:**
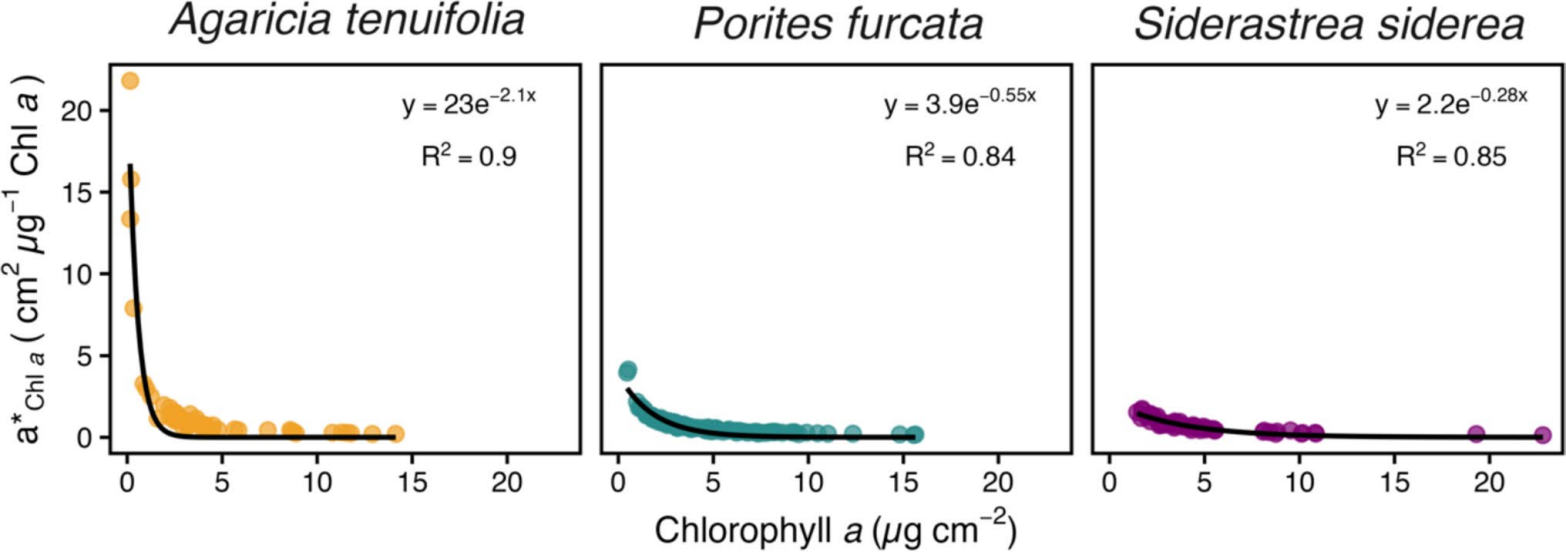
Absorption efficiency (a*) of chlorophyll *a* (Chl *a*) for *Agaricia tenuifolia* (yellow), *Porites furcata* (teal) and *Siderastrea siderea* (purple). Each point represents a coral fragment, with a* calculated as absorbance normalized to Chl *a *content (µg cm^-2^). The fitted exponential models, equations, and coefficient of determination are presented.

The non-linearity of the relationship between NDVI and Chl *a* of scleractinian corals stands in contrast to the linear association observed in the soft coral *Sacrophyton* cf. *glaucum* (Leal et al. 2015), although the limited number of high-chlorophyll data points in that study constrains the strength of their linear inference. We propose that this non-linearity is caused by the optical properties of the coral skeleton, which scatters light and influences the reflectance signal which is itself affected by changes in pigment density (Enríquez et al. 2005; Terán et al. 2010; Wangpraseurt et al. 2017). Across species, the absorption coefficient of Chl *a* (a*) increased as Chl *a* concentrations declined (Fig. 4), indicating more efficient light absorption per unit pigment at lower pigment densities. The modeled declines showed the steepest decrease in *A. tenuifolia* (87.8% per unit Chl *a*), followed by *P. furcata* (42.3%) and *S. siderea* (24.3%). These differences suggest species-specific sensitivity in how pigment concentration influences absorption efficiency. As light passes more easily through the coral tissue due to the decreasing pigment content, it is increasingly reflected by the calcium carbonate skeleton. The reflected light thus passes through the pigment layer multiple times, disproportionately increasing the overall absorption by the remaining pigments. As a result, absorbance, and consequently reflectance and NDVI, change relatively less at low densities, because the remaining pigments compensate through enhanced absorption efficiency (Terán et al. 2010). This compensation leads to a slower decline in NDVI values at lower Chl *a* concentrations during bleaching, and an overestimation of actual Chl *a* content.

It is important to note that non-linearity also occurred at high Chl *a* concentrations. Small initial declines in symbiont/pigment density likely do not induce measurable differences in reflectance, due to high levels of symbiont self-shading in heavily pigmented, unbleached corals (Anthony et al. 2005). Simulation studies found that initial reductions in symbiont density of up to 20% from healthy, unbleached states with 10^6^ symbiont cells cm^−2^, do not lead to a proportional reduction in reflectance (Terán et al. 2010). As a result, linear NDVI models may initially underestimate Chl *a* loss. Thus, when analyzed assuming a linear instead of a non-linear relationship between reflectance-NDVI and Chl *a*, gradually declining NDVI values may give the false impression of an inflated healthy state (i.e., an overestimation of Chl *a*, or underestimation of bleaching progression) at both ends of the pigmentation spectrum.

Variation in the NDVI-Chl *a* relationship across coral species likely reflects differences in skeletal morphology and structure, which govern the degree of light enhancement and internal scattering experienced by the symbionts (Marcelino et al. 2013; Enríquez et al. 2017; Wangpraseurt et al. 2017; Swain et al. 2018). Swain et al. (2016) demonstrated that skeletal light-scattering accelerates bleaching responses in reef-building corals, with species exhibiting higher skeletal light-scattering showing increased bleaching susceptibility. Massive species like *S. siderea* scatter less light and show smaller changes in absorption efficiency, whereas branching species, such as *P. furcata*, enhance internal light more efficiently (Marcelino et al. 2013; Enríquez et al. 2017), resulting in a stronger non-linear relationship between NDVI and Chl *a*. Sheet-like morphologies, like that of *A. tenuifolia,* may transmit light with little internal scattering, contributing to a more linear pattern. These differences indicate that species-specific skeletal light scattering traits influence reflectance-based NDVI measurements and should be considered when interpreting pigment proxies across taxa.

In addition to skeletal optical effects, several biological and technical factors may influence reflectance-based Chl *a* estimates across species. Biological sources of variation include tissue thickness, the distribution of symbionts and Chl within tissues, and the presence of host pigments such as chromoproteins and fluorescent proteins, as well as endolithic algae within the skeleton (Bollati et al. 2020; Galindo-Martínez et al. 2022). Specifically, upregulation of chromo- or fluorescent proteins dampen skeletal backscattering, thereby potentially making the Chl *a*-NDVI relationship more linear. Photoacclimation can generate substantial intraspecific variation by affecting symbiont composition and Chl concentration (e.g., Lesser et al. 2010; Frade et al. 2008a, b, c), and can also drive long-term changes in skeletal morphology (Einbinder et al. 2009). These factors may influence local reflectance properties and, consequently, NDVI measurements, possibly representing a significant source of intraspecific variation. Nonetheless, our calibration results demonstrate that NDVI provides a robust proxy for Chl *a* within the conditions examined here.

In conclusion, we confirm that NDVI is a reliable proxy for Chl *a* content in scleractinian corals, enabling rapid, non-invasive, cost-effective, and high-resolution monitoring of coral responses to thermal and light stress. To account for the observed non-linear, likely exponential relationship, we recommend to log-transform Chl *a* and to establish species-specific calibration curves that accurately capture differences in skeletal optical properties and absorption coefficients. With spectrometers and detailed protocols for measuring NDVI (Watty et al. 2024) becoming more widely available, reflectance-based phenotyping tools have the potential to be established as standardized methods for measuring coral physiological conditions ex- and in situ (see Naugle et al. 2024, 2025; Denis et al. 2024). This approach is not limited to corals and could be applied to other photosymbiotic or photosynthesizing marine taxa, such as symbiotic bivalves or crustose coralline algae. Moreover, additional spectral markers could be developed to assess the health of various marine organisms, as proposed by Teague et al. (2022). Beyond local measurements, NDVI has potential for remote sensing applications, including monitoring reef productivity or bleaching events (see Veeranjaneyulu et al. 2024).

## Supplementary Information

Below is the link to the electronic supplementary material.

Supplementary file1 (XLSX 4469 KB)

Supplementary file2 (DOCX 763 KB)

## Data Availability

Data is provided within the manuscript or supplementary information files.
