# Supplementary material for: Using reflectance to measure chlorophyll a in corals: calibration and implications of skeletal optical properties
Source: Coral Reefs. 2026 Feb 17;45(3):1495–503. doi: 10.1007/s00338-026-02831-0 (PMC13282203; doi:10.1007/s00338-026-02831-0)
Supplement: Supplementary file 2 — Supplementary file2 (DOCX 763 KB) [file 338_2026_2831_MOESM2_ESM.docx]

Supplementary Materials for

**Using reflectance to measure chlorophyll *a* in corals: calibration and implications of skeletal optical properties**

Coral Reefs

Kay Watty^1,*^, Verena Schoepf^1^, Kelly W. Johnson^1^, Sophie Littke^1,2^, Rene M. van der Zande^1^

^1^Department of Freshwater and Marine Ecology, Institute for Biodiversity and Ecosystem Dynamics, University of Amsterdam, Amsterdam, The Netherlands

^2^University of Algarve, Faro, Portugal

*Correspondence: khw@watty.eu

**Supplementary Methods**

Outliers were assessed for potential influence on the regression models using z-scores, Mahalanobis distance, and Cook’s distance. Points with Cook’s distance below 1 were considered to have marginal influence on the regression and retained in the analysis (Cook and Weisberg 1982). For points with a Cook’s distance between 0.5 and 1, we also evaluated their influence by performing the analysis without them.

**Supplementary Results**

Spearman and Pearson correlation analyses revealed a significant positive association between NDVI and Chl *a* content across all species, though the strength and type of relationship varied (Table S1). *Agaricia tenuifolia* showed strong monotonic and linear relationships (Spearman’s $\rho$ = 0.71, Pearson’s r = 0.75). *Porites furcata* exhibited the strongest monotonic correlation ($\rho$ = 0.82) and a slightly weaker linear association (r = 0.73), suggesting non-linearity in the relationship. *Siderastrea siderea* demonstrated moderate correlations ($\rho$ = 0.49, r = 0.59), indicating a weaker overall association between NDVI and Chl *a* in this species. These results highlight a consistent but species-specific association between spectral reflectance derived NDVI and Chl *a* content.

To further characterize this relationship, three models were evaluated: a linear regression, a linear regression on log-transformed Chl *a*, and an exponential model (Fig. 3, Table S2). The linear model on log-transformed Chl *a* consistently provided the best fit across species. For *A. tenuifolia*, it achieved an R^2^ of 0.625 and an AIC of 54, providing a better fit than the exponential (R^2^ = 0.656, AIC = 224) and linear models (R^2^ = 0.559, AIC = 236) in terms of information criterion, despite slightly lower R^2^ than the exponential model. Similarly, for *P. furcata,* the linear model on log-transformed Chl *a* had the highest R^2^ (0.663) and lowest AIC (56), indicating a substantially better fit than the exponential (R^2^ = 0.565, AIC = 485) or linear model (R^2^ = 0.531, AIC = 494). In *S. siderea*, all models performed weaker overall. The exponential model had the highest R^2^ (0.458), but the linear model on log-transformed Chl *a* had a far lower AIC (61) compared to the linear (AIC = 296) and exponential models (AIC = 285), indicating a better balance between model complexity and fit despite a lower R^2^ (0.363). The linear model performed worst (R^2^ = 0.342). The linear models on log-transformed Chl *a* consistently outperformed the other models across species, suggesting that the relationship between NDVI and Chl *a* is better captured when log-transforming Chl *a*, likely due to underlying non-linear patterns.

Shapiro-Wilk tests of model residuals supported these findings (Table S3). The linear models on log-transformed Chl *a* passed the normality test for *A. tenuifolia* (p = 0.456) and *P. furcata* (p = 0.111), while the linear and exponential models showed significant deviations (p < 0.05). In *S. siderea*, all models failed the test (p < 0.05), but the linear model on log-transformed Chl *a* had the least deviation (p = 0.013).

Diagnostic plots further corroborated these trends (Supplementary Figs. S1-S3). Linear models for *A. tenuifolia* and *P. furcata* exhibited heteroscedasticity and non-normality, which improved substantially in the linear model on log-transformed Chl *a*. For *S. siderea*, deviations from normality and the presence of influential points in the linear and exponential models were reduced in the linear model on log-transformed Chl *a*. Two data points at the upper range of chlorophyll concentration for *S. siderea* were flagged as extreme outliers in the linear regression based on z-score analysis and Mahalanobis distance, and had a Cook’s distance of about 0.5. Removal of these points did not change the significance of the regression (p < 0.001), but R^2^ decreased slightly from 0.34 (with outliers) to 0.27 (without). Because the Cook’s distances were below the commonly used threshold of 1 (Cook and Weisberg 1982), the values did not result from measurement error and fall within the expected biological range (e.g., Torres-Pérez et al. 2015; Bove et al. 2022; Radice et al. 2023), the outliers were not excluded from the analysis. In summary, the log-linear model consistently outperformed both the linear and exponential models across species. It offered a more robust fit, minimized assumption violations, and provided stronger support for a non-linear, potentially exponential, relationship between NDVI and Chl *a* in scleractinian corals.

1. **Supplementary Tables**

**Table S1** Correlation tests per coral species

| **Species** | **Spearman** | | **Pearson** | |
| --- | --- | --- | --- | --- |
|  | $\boldsymbol{\rho}$ | ***p*** | **r** | ***p*** |
| ***Agaricia tenuifolia*** | 0.707 | < 0.001 | 0.748 | < 0.001 |
| ***Porites furcata*** | 0.817 | < 0.001 | 0.728 | < 0.001 |
| ***Siderastrea siderea*** | 0.491 | < 0.001 | 0.585 | < 0.001 |

**Table S2** Regression model comparisons per coral species.

| **Species** | **Model** | **R^2^** | **AIC** | **F (df)** | ***p*** |
| --- | --- | --- | --- | --- | --- |
| ***Agaricia tenuifolia*** | linear | 0.559 | 236 | 62.2 (1, 49) | < 0.001 |
|  | log-linear | 0.625 | 54 | 81.8 (1, 49) | < 0.001 |
|  | exponential | 0.656 | 224 | - | - |
| ***Porites furcata*** | linear | 0.531 | 494 | 124.4 (1, 110) | < 0.001 |
|  | log-linear | 0.663 | 56 | 216 (1, 110) | < 0.001 |
|  | exponential | 0.565 | 485 | - | - |
| ***Siderastrea siderea*** | linear | 0.342 | 296 | 28.1 (1, 54) | < 0.001 |
|  | log-linear | 0.363 | 61 | 30.8 (1, 54) | < 0.001 |
|  | exponential | 0.458 | 285 | - | - |

**Table S3** Shapiro-Wilk p-values* per regression model and species

| **Species** | **Linear** | **Log-linear** | **Exponential** |
| --- | --- | --- | --- |
| ***Agaricia tenuifolia*** | 0.014 | 0.456 | < 0.001 |
| ***Porites furcata*** | < 0.001 | 0.111 | < 0.001 |
| ***Siderastrea siderea*** | < 0.001 | 0.013 | < 0.001 |

*p-values < 0.05 indicate a significant deviation from normality

**Table S4** Functions of exponential and log-linear model fits

| **Species** | **Exponential** | **Back transformed log-linear** |
| --- | --- | --- |
| ***Agaricia tenuifolia*** | $y=0.692 e^{4.39 x}$ | $y= e^{0.21 + 3.42 x}-1$ |
| ***Porites furcata*** | $y=0.93 e^{3.98 x}$ | $y= e^{0.42 + 3.16 x}-1$ |
| ***Siderastrea siderea*** | $y=1.02 e^{3.42 x}$ | $y= e^{0.77 + 2.1 x}-1$ |

1. **Supplementary Figures**

**Fig. S1** Diagnostic plots for *Agaricia tenuifolia* showing residual analyses for the linear, log-linear, and exponential models

**Fig. S2** Diagnostic plots for *Porites furcata* showing residual analyses for the linear, log-linear, and exponential models

**Fig. S3** Diagnostic plots for *Siderastrea siderea* showing residual analyses for the linear, log-linear, and exponential models

**Supplementary References**

Bove CB, Davies SW, Ries JB, Umbanhowar J, Thomasson BC, Farquhar EB, McCoppin JA, Castillo KD (2022) Global change differentially modulates Caribbean coral physiology. Plos One 17(9):e0273897. https://doi.org/10.1371/journal.pone.0273897

Cook RD, Weisberg S (1982) Residuals and Influence in Regression. New York: Chapman & Hall

Radice VZ, Martinez A, Paytan A, Potts DC, Barshis DJ (2024) Complex dynamics of coral gene expression responses to low pH across species. Molecular Ecology (1):e17186. https://doi.org/10.1111/mec.17186

Torres-Pérez JL, Guild LS, Armstrong RA, Corredor J, Zuluaga-Montero A, Polanco R (2015) Relative pigment composition and remote sensing reflectance of Caribbean shallow-water corals. PLoS One 10(11):e0143709. https://doi.org/10.1371/journal.pone.0143709
